# A Laboratory Text-Book of Pathology, for the Use of Students and Practitioners of Medicine

**Published:** 1898-06

**Authors:** 


A LABORATORY TEXT-BOOK OF PATHOLOGY, for the Use of Students and Practitioners of Medicine. By HORACE J. WHITACRE, B. S., M. D., Demonstrator of Pathology in the Medical College of Ohio. 121 illustrations. Philadelphia: P. Blakiston, Son & Co. 1898.

One of the best laboratory guides for the student yet issued is the manual of Whitacre, which gives the essentials of pathology and leaves the generalities for the larger treatises. It deals with the various pathological conditions of the internal organs, inflammation, the degenerations, tumors and infective granulomata. The different conditions are illustrated by photomicrographs, which enable the student to see the tissue as it really is, and not as the author thinks he ought to see it. These photomicrographs are all well chosen, are truthful in their delineation and form the bulk of excellence of this work. They are well worth the price of the manual.

